# Towards an *In Vitro* Model of *Plasmodium* Hypnozoites Suitable for Drug Discovery

**DOI:** 10.1371/journal.pone.0018162

**Published:** 2011-03-31

**Authors:** Laurent Dembele, Audrey Gego, Anne-Marie Zeeman, Jean-François Franetich, Olivier Silvie, Armelle Rametti, Roger Le Grand, Nathalie Dereuddre-Bosquet, Robert Sauerwein, Geert-Jan van Gemert, Jean-Christophe Vaillant, Alan W. Thomas, Georges Snounou, Clemens H. M. Kocken, Dominique Mazier

**Affiliations:** 1 Université Pierre et Marie Curie-Paris 6, UMR S945, Paris, France; 2 Institut National de la Santé et de la Recherche Médicale U945, Paris, France; 3 Department of Parasitology, Biomedical Primate Research Centre, Rijswijk, The Netherlands; 4 Division of Immuno-Virology, Institute of Emerging Diseases and Innovative Therapies (IMETI), Commissariat à l'Energie Atomique (CEA), Fontenay-aux-Roses, France; 5 Université Paris-Sud XI, UMR-E01, Orsay, France; 6 Department of Medical Microbiology, Radboud University Nijmegen Medical Centre, Nijmegen, The Netherlands; 7 Service de Chirurgie Digestive, Hépato-Bilio-Pancréatique et Transplantation Hépatique, Hôpital Pitié-Salpêtrière, Paris, France; 8 AP-HP, Groupe Hospitalier Pitié-Salpêtrière, Service Parasitologie-Mycologie, Paris, France; Agency for Science, Technology and Research - Singapore Immunology Network, Singapore

## Abstract

**Background:**

Amongst the *Plasmodium* species in humans, only *P. vivax* and *P. ovale* produce latent hepatic stages called hypnozoites, which are responsible for malaria episodes long after a mosquito bite. Relapses contribute to increased morbidity, and complicate malaria elimination programs. A single drug effective against hypnozoites, primaquine, is available, but its deployment is curtailed by its haemolytic potential in glucose-6-phosphate dehydrogenase deficient persons. Novel compounds are thus urgently needed to replace primaquine. Discovery of compounds active against hypnozoites is restricted to the *in vivo P. cynomolgi*-rhesus monkey model. Slow growing hepatic parasites reminiscent of hypnozoites had been noted in cultured *P. vivax*-infected hepatoma cells, but similar forms are also observed *in vitro* by other species including *P. falciparum* that do not produce hypnozoites.

**Methodology:**

*P. falciparum* or *P. cynomolgi* sporozoites were used to infect human or *Macaca fascicularis* primary hepatocytes, respectively. The susceptibility of the slow and normally growing hepatic forms obtained *in vitro* to three antimalarial drugs, one active against hepatic forms including hypnozoites and two only against the growing forms, was measured.

**Results:**

The non-dividing slow growing *P. cynomolgi* hepatic forms, observed *in vitro* in primary hepatocytes from the natural host *Macaca fascicularis*, can be distinguished from similar forms seen in *P. falciparum*-infected human primary hepatocytes by the differential action of selected anti-malarial drugs. Whereas atovaquone and pyrimethamine are active on all the dividing hepatic forms observed, the *P. cynomolgi* slow growing forms are highly resistant to treatment by these drugs, but remain susceptible to primaquine.

**Conclusion:**

Resistance of the non-dividing *P. cynomolgi* forms to atovaquone and pyrimethamine, which do not prevent relapses, strongly suggests that these slow growing forms are hypnozoites. This represents a first step towards the development of a practical medium-throughput *in vitro* screening assay for novel hypnozoiticidal drugs.

## Introduction

In mammalian hosts, malaria infection is initiated by the inoculation of *Plasmodium* sporozoites by an infected anopheline mosquito. These parasite forms then make their way to the liver where they specifically invade hepatocytes, the host cells permissive to full maturation leading to the production of merozoites that initiate the erythrocytic cycle. In most *Plasmodium* species, invasion of the hepatocyte by the sporozoite proceeds seamlessly to the development of the hepatic parasite culminating in the release of merozoites into the blood stream. The minimum time between the infective bite and the blood infection varies with the parasite species (2 to 15 days). However, in five parasite species, three in southeast Asian monkeys (*P. cynomolgi*, *P. fieldi* and *P. simiovale*) and two in humans (*P. vivax* and *P. ovale*), a subset of the sporozoites that have invaded the hepatocyte do not develop beyond the early hepatic trophozoite stage, but remain in a state of latency in which they can persist for many years [1], [2]. These forms, called hypnozoites, resume their development to generate infective merozoites many weeks, months or years later, though the timing and trigger for this “re-activation” remains mysterious. The hypnozoites probably provide a significant evolutionary advantage, particularly in temperate climates, as they effectively extend the duration of infection and, thus the opportunities to transmit successfully via mosquitoes to new hosts.

The demonstration of hypnozoites in *P. cynomolgi*-infected rhesus macaques in the early 1980's provided an explanation for the relapses observed during infections with these five parasite species [3]. A relapse is a blood stage episode that can occur after full clearance of parasites from the erythrocytic parasites (naturally or via treatment) from a primary or a recrudescent episode. In *P. vivax*, relapses are often observed regularly over many months and even years after the infectious mosquito bite. A similar pattern is also observed for *P. ovale*. Moreover, some temperate *P. vivax* strains produce sporozoites that nearly all transform into hypnozoites such that the primary blood infection is only observed many months after the infective mosquito bite, when some of the hypnozoite reactivate [1], [2]. Given that *P. vivax* is the most widely distributed of the four species of malaria parasites specific to humans and the most prevalent, except for sub-Saharan Africa where *P. falciparum* dominates and *P. ovale* infections are common [4], the occurrence of hypnozoites/relapses adds to their morbidity burden and makes these species especially difficult to eradicate.

Only one antimalarial drug capable of eliminating all liver stages including hypnozoites, the 8-aminoquinoline primaquine, is currently available [5], [6]. Its short half-life in the serum necessitates a lengthy treatment regimen that is not conducive to good adherence. Another recently developed 8-aminoquinoline with a longer half-life, tafenoquine, is equally effective against hypnozoites. However, the major shortcoming of primaquine and tafenoquine is that administration to persons with glucose-6-phosphate dehydrogenase deficiency (G6PD) is associated with a high risk of severe haemolytic anaemia. This precludes their widespread use because carriage of this genetic trait is common in the majority of the populations in *P. vivax* and *P. ovale* endemic areas.

The revival of eradication as the ultimate goal of malaria control programmes and the rediscovery that the clinical and socio-economic burdens of vivax malaria rival those of falciparum malaria, makes it imperative to develop novel compounds able to eliminate hypnozoites. Such a drug discovery programme cannot be currently envisaged simply because the only model available to screen for activity against hypnozoites is the *P. cynomolgi* sporozoite infection in rhesus monkeys. This host-parasite combination, whose development was initiated in 1944, remains the acknowledged biological and chemotherapeutic model for *P. vivax* infections, including erythrocytic and hepatic stages [7], [8], [9], [10]. However, an *in vitro* model is necessary in order to screen large numbers of new compounds for hypnozoiticidal activity. Hopes to achieve this were raised with the establishment of *in vitro* cultured hepatic stages of *P. vivax* [11], [12] and then *P. cynomolgi* [13], [14] in their respective primary host hepatocytes. In parallel, *P. vivax* hepatic stage maturation was obtained in the hepatoma cell line HepG2-A16 [15]. Observation of non-dividing *P. vivax* forms in HepG2-A16 cells when most hepatic forms have developed to form mature schizonts (5–8 days after sporozoite inoculation), prompted the suggestion that these corresponded to hypnozoites [15]. This was re-enforced when the proportion of these non-dividing forms correlated with the expected proportion of hypnozoites to dividing hepatic parasites for different *P. vivax* strains, namely in equal numbers from tropical strains and with a predominance of hypnozoites from temperate strains [16], [17]. However, these morphological observations alone are of insufficient weight to establish the actual nature of the non-dividing forms observed as hypnozoites. Failure of hepatic parasites to reach to first mitotic division might be due to other factors, such as defective sporozoites, or failure to thrive in a metabolically unsuitable host cell, or in a sub-optimal culture environment. Indeed, cultured hepatic parasites, as well as those found *in vivo* under natural conditions of infection, are often asynchronous, with large variations in size and developmental stage as cultivation progresses. Furthermore, in our experience small uninuclear hepatic forms are equally regularly observed along with mature hepatic schizonts in primary hepatocyte or hepatoma cell cultures infected with sporozoites from *Plasmodium* species that do not form hypnozoites, such as *P. falciparum* or *P. berghei*.

We have recently proposed that the differential susceptibility of normal hepatic forms and of hypnozoites to defined anti-malarial drugs might serve to indicate the presence of hypnozoites in infected hepatocyte cultures [18]. This was tested in parallel cultures of *P. falciparum* and *P. cynomolgi* infections in their host's respective primary hepatocytes, because this provides a means to establish if the non-dividing parasites obtained from the relapsing species (*P. cynomolgi*) and the non-relapsing species (*P. falciparum*) are biologically distinct.

## Results and Discussion

### Slow growing and normally developing hepatic forms occur in hepatocyte cultures

Primary hepatocytes were isolated from liver segments obtained from humans or from *Macaca fascicularis*, the natural host of *P. cynomolgi* [1], [2]. The primary hepatocyte cultures were infected with *P. falciparum* or *P. cynomolgi* sporozoites respectively, and then maintained for 11 days. Each day some wells from the cultures were fixed and the numbers and sizes of the hepatic forms present were measured. The hepatic forms were visualized using two stains: DAPI that reveals the nuclei and an antibody raised against a *P. falciparum* heat shock protein 70.1 (HSP70.1) [19], which recognizes both *P. falciparum* and *P. cynomolgi* parasites. HSP70.1 expression in hepatic stages is initiated about three hours after hepatocyte invasion and is maintained until full maturation of the hepatic schizont.

**Figure 1:**
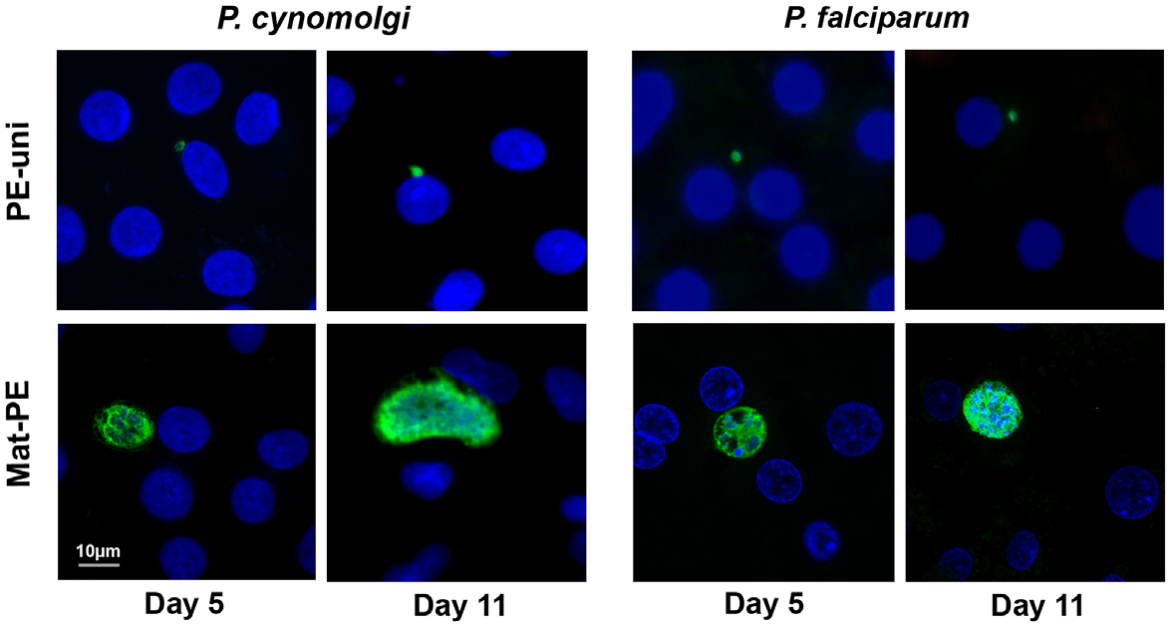
Slow growing and normally developing pre-erythrocytic (PE) forms. Two types of hepatic parasites can be clearly distinguished from days 5 post-infection onwards, in *in vitro* cultured human or *M. fascicularis* primary hepatocytes infected with *P. falciparum* or *P. cynomolgi* sporozoites, respectively. The representative photomicrographs were made on cultures fixed on Day 5 and Day 11 post-infection. The parasite and host nuclei are stained with DAPI (blue), while the parasites are labelled by an antibody specific to the HSP70 of the two parasite species (green). Mature PE forms (Mat-PE) and uninucleate small PE forms (PE-uni) are clearly distinguishable.

**Figure 2:**
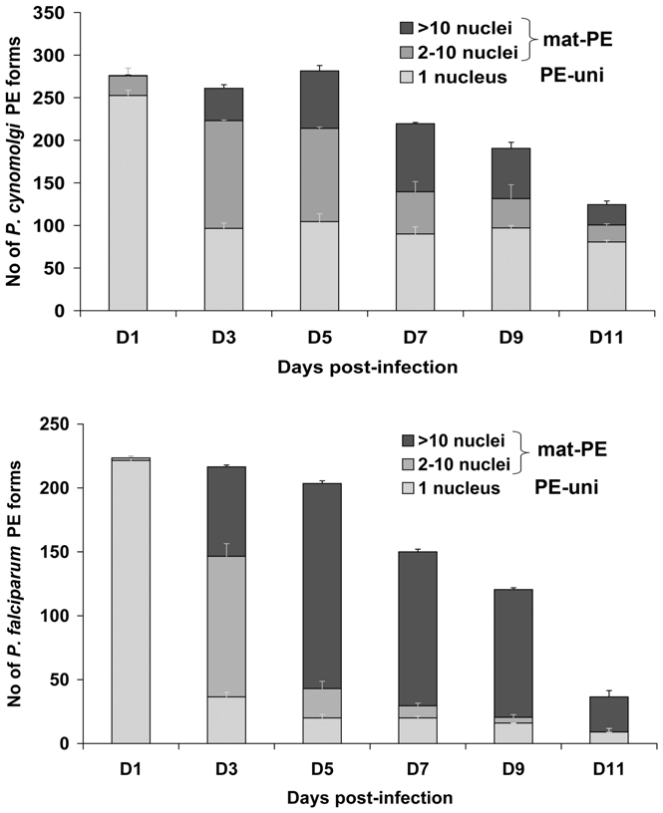
Maturation dynamics of cultured *P. falciparum* and *P. cynomolgi* hepatic forms. The proportion of PE forms at different stages of maturation (as assessed by the extent of nuclear division) is presented for cultures of increasing age. Uninucleate parasite forms correspond to the small forms (PE-uni) shown in Figure 1, while all others correspond to the mat-PE forms. The data was representative of three independent experiments.

As expected, from Day 3 post-inoculation onwards, most *P. falciparum* and about half of *P. cynomolgi* hepatic forms obtained developed normally (parasite nuclear division and size increase), however in a proportion of the infected hepatocytes the parasites remained uninucleate (Fig. 1) and did not grow beyond their size on Day 1. The smaller uninucleate pre-erythrocytic (PE) forms were termed PE-uni, while the maturing forms were termed mat-PE. As the infection progressed, some differences were observed between the *P. falciparum* and *P. cynomolgi* cultures (Fig. 2). Nearly 40% of the hepatic forms in the *P. cynomolgi* were of the PE-uni type on Day 3 and these forms remained unchanged by Day 11, at which time they represented nearly two thirds of the hepatic forms as by that time nearly all developing forms had fully matured and burst. In contrast, the *P. falciparum* PE-uni forms were fewer in numbers from the outset and although their absolute numbers remained constant between Day 5 and Day 9, they had significantly declined by Day 11 (*p*<0.05, Mann-Whitney test). When both cultures were stopped on Day 11, most of the remaining mat-PE forms were large mature hepatic schizonts each with a large number of visible parasite nuclei. Similar proportions of long-lived PE-uni forms were obtained when *P. cynomolgi* sporozoites were used to infect cultured rhesus monkey (*M. mulatta*) primary hepatocytes (data not shown).

### Differential activity of selected antimalarial drugs distinguished the two hepatic forms

The PE-uni observed in the *P. falciparum* (a non-hypnozoite producing species) and in the *P. cynomolgi* (a hypnozoite producing species) hepatic cultures are indistinguishable from the non-dividing forms postulated to be hypnozoites that were observed in cultured HepG2 cells infected with *P. vivax* strains of different geographical origins [15], [16], [17], [20].

In order to provide unequivocal proof that the PE-uni forms observed in the *P. cynomolgi* (or *P. vivax*) hepatic cultures are indeed hypnozoites, it would be necessary to observe after a suitable period of latency (at least 3–4 weeks), a reactivation of the maturation process that would lead to the release of merozoites. This would be difficult for HepG2 cultures because beyond a week of incubation, the multiplication of the hepatoma cells will irrevocably lead to an unacceptable dilution of the few non-dividing forms. This can be overcome by prior irradiation of the HepG2 cells prior to inoculation with sporozoites, though it is not clear that hypnozoites are capable of forming and/or being reactivated in hepatoma cells. Primary hepatocytes do not divide in culture, but maintaining their viability beyond a few weeks has proven difficult.

Given these limitations, we postulated that data consistent with a hypnozoite identity for the *P. cynomolgi* PE-uni forms could be derived by exploiting the contrasting activities of three current anti-malarial drugs against hypnozoites [18]. Pyrimethamine (an inhibitor of dihydrofolate reductase), atovaquone (an inhibitor of the mitochondrial cytochrome c) and primaquine (putatively a mitochondrial inhibitor), have all been demonstrated to fully inhibit normal hepatic stage parasites *in vivo* and *in vitro* [10], [21], [22], [23], [24], [25]. Only primaquine however, is able additionally to eliminate hypnozoites [5], [6].

**Figure 3:**
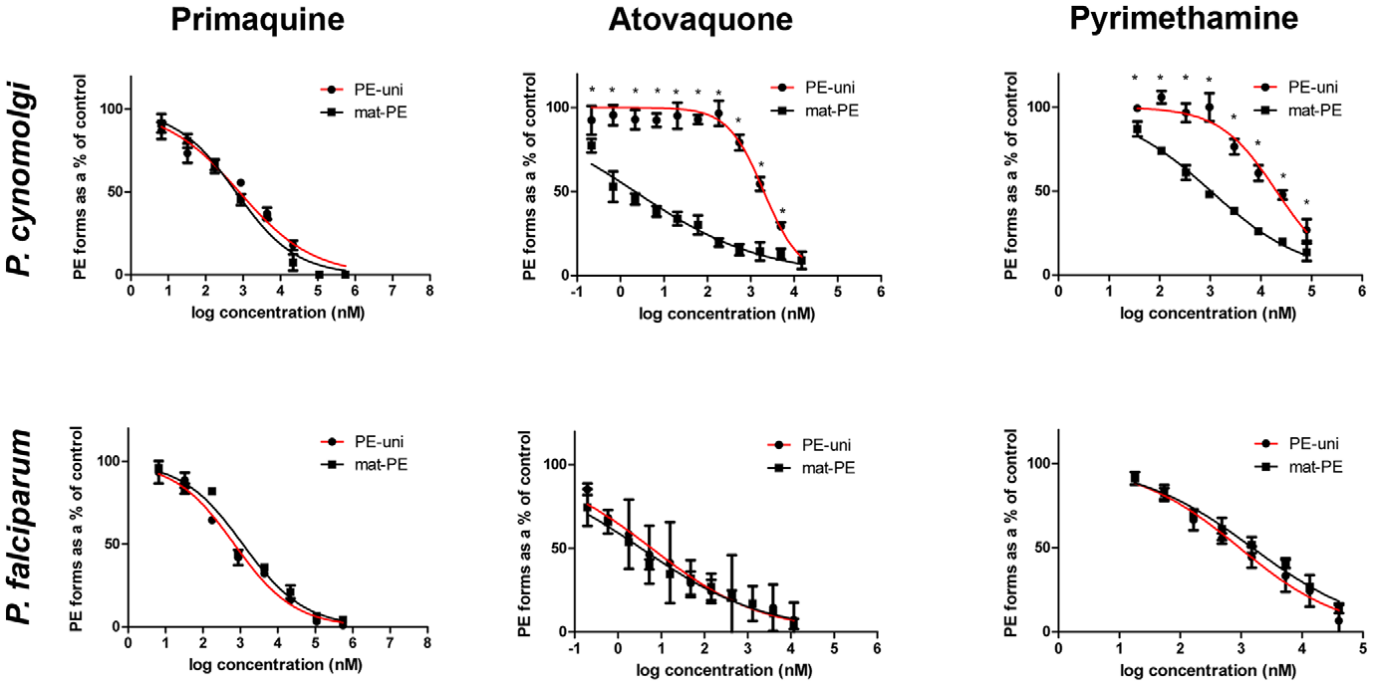
Dose-response curves for primaquine, atovaquone and pyrimethamine against the distinct hepatic forms observed for *P. falciparum* and *P. cynomolgi*. The infected primary hepatocyte cultures were exposed to the varying drug concentrations from Day 5 to Day 8 post-inoculation and fixed on D8. The curves obtained for the PE-uni forms are presented in red, while those for the Mat-PE forms are presented in black. The data was representative of two independent assays for pyrimethamine (for both parasite species) and for primaquine (*P. falciparum*); and of three independent experiments for atovaquone (both parasite species) and primaquine (*P. cynomolgi*). *, p<0.05.

Having established above that the PE-uni forms are most evident between Day 5 and Day 9 of cultivation, during which time their numbers remain stable for both *P. falciparum* and *P. cynomolgi*, we obtained the IC_50_ values for these drugs against the PE-uni and the mat-PE forms following exposure to the drugs for three days from Day 5 to Day 8 (Fig. 3 and Table 1). As expected, the two types of hepatic forms (mat-PE and PE-uni) from both parasite species were eliminated with primaquine, with similar IC_50_ values (650 nM to 1166 nM, equivalent to 0.3 µg/ml–0.53 µg/ml of the diphosphate salt). In all cases, full inhibition was observed as the concentration reached 110 µM (50 µg/ml), though it should be noted that *M. fascicularis* hepatocytes viability decreased with exposure to primaquine concentrations higher than 22 µM (10 µg/ml) as assessed by MTT (TC_50SH_ = 56 µM). By contrast, while both pyrimethamine and atovaquone inhibited the development of the mat-PE forms of both parasite species as well as the non-dividing PE-uni of *P. falciparum*, the *P. cynomolgi* PE-uni forms showed resistance to these drugs, displaying a 900-fold and a 20-fold increase in the IC_50_ values for atovaquone and pyrimethamine, respectively.

**Table 1:** IC_50_ values for selected drugs against the different hepatic parasites.

	Primaquine IC_50_ in µM (95% CI)		Atovaquone IC_50_ in nM (95% CI)		Pyrimethamine IC_50_ in µM (95% CI)	
	PE-uni	mat-PE	PE-uni	mat-PE	PE-uni	mat-PE
***P. cynomolgi***	**0.80** (0.55–1.17)	**0.65** (0.50–0.85)	**1997** (1638–2435)	**2.13** (1.38–3.30)	**20.31** (14.95–27.60)	**1.03** (0.84–1.25)
***P. falciparum***	**0.69** (0.51–0.94)	**1.17** (0.80–1.70)	**6.09** (2.67–13.94)	**3.48** (2.21–.47)	**0.94** (0.65–.37)	**1.53** (1.18–1.99)

The inhibition of the PE-uni forms by primaquine indicates that they are metabolically active. However, the resistance of the *P. cynomolgi* PE-uni forms to atovaquone and pyrimethamine, as compared to the sensitivity of the *P. falciparum* PE-uni forms to these drugs, suggests that the metabolism of the cultured non-dividing hepatic parasites differs between the two species. The concordance of these observations *in vitro* with the known inability of pyrimethamine and atovaquone to prevent relapses and thus, eliminate hypnozoites *in vivo*, strongly suggests that the majority of the *P. cynomolgi* PE-uni forms we observed in culture represent hypnozoites.

**Figure 4:**
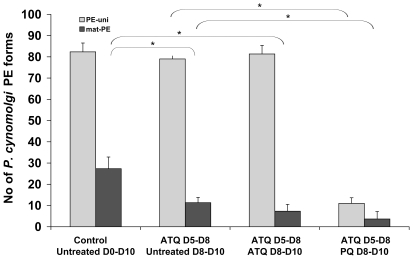
Selection of *P. cynomolgi* PE-uni forms prior to screening for hypnozoiticidal activity. Cultures were treated with atovaquone (ATQ) at 67 ng/ml (182 nM) for three days starting on Day 5 post-inoculation in order to eliminate the mat-PE parasites. Subsequently the cultures were either left untreated, treated for a further two days with ATQ (as above), or primaquine (PQ) at 10 µg/ml (22 nM). The cultures were then stopped by methanol fixation on Day 10 and the hepatic forms present enumerated. The data was representative of three independent experiments. *, *p*<0.05.

Since the ultimate aim is to develop a viable screening assay to identify novel hypnozoitocidal drugs, we modified the assay to include a PE-uni selection step before testing for primaquine activity against these forms. This was achieved by treating *P. cynomolgi* hepatic cultures with atovaquone between Day 5 and Day 8. Subsequent to this, most of the hepatic parasites present in the cultures were the PE-uni forms, and these forms persisted unaffected whether atovaquone treatment was withdrawn or maintained another three days, up to Day 10. By contrast, their numbers were significantly diminished by exposure to primaquine on Day 8 to Day 10 (Fig. 4).

### Conclusions

Here we demonstrate that the non-dividing *P. cynomolgi* and *P. falciparum* hepatic forms that are observed in cultured primary hepatocytes isolated from their respective natural hosts are distinct. The higher PE-uni numbers observed for *P. cynomolgi*, their maintenance in undiminished numbers after 10 days of cultivation, and their resistance to inhibition by atovaquone and pyrimethamine distinguishes them from the *P. falciparum* PE-uni forms. These observations, when associated to susceptibility to elimination by primaquine and to the contrasting relapse patterns of sporozoite-induced *P. cynomolgi* infections in rhesus monkeys after primaquine or pyrimethamine treatment, provide compelling support for the hypothesis that these *in vitro* PE-uni forms actually represent hypnozoites. In any case, the PE-uni forms seem to provide a hypnozoite surrogate suitable for drug discovery programs. Given the long-established biological and chemotherapeutic equivalence of *P. vivax* and *P. cynomolgi* infections [8], [9], [10], we consider it likely that similar PE-uni forms will be observed for *P. vivax*. It would also be reasonable to suggest that the non-dividing *P. vivax* forms initially observed in HepG2-A16 cells in mid 1980 [15], [16] and very recently [20] could also represent hypnozoites. Nonetheless, this would need a validation similar to the one presented here because it is not known whether hepatoma cells lines present a suitable environment for hypnozoite formation and eventual reactivation.

The work described here represents a first significant step towards the development of a screening assay aimed at the discovery of novel hypnozoiticidal compounds. The use of *P. cynomolgi* for this endeavour is justified by the fact that the requisite provision of a constant supply of sporozoites from the same strain, or eventually clone, can be achieved without the necessity to infect chimpanzees as is the case for *P. vivax* [20], or rely on patients infected by different *P. vivax* strains. On the other hand, if medium-throughput screening programs are envisaged it would be necessary to have access to easy-to-culture cell lines that are at least as susceptible to sporozoite infection as the primary hepatocytes isolated from monkey liver segments. Finally, the availability of a potential *in vitro* hypnozoite model opens the way to conduct detailed investigations into the nature and mechanisms of latency in *Plasmodium* parasites, a hitherto closed avenue of fundamental biological interest.

## Methods

### Ethics Statement

Human hepatocytes were isolated from liver segments taken after oral informed consent from adult patients undergoing partial hepatectomy as part of their medical treatment (Service de Chirurgie Digestive, Hépato-Bilio-Pancréatique et Transplantation Hépatique, Hôpital Pitié-Salpêtrière, Paris, France). The collection and use of this material for the purposes of the study presented here were undertaken in accordance with French national ethical guidelines under Article L. 1121-1 of the “Code de la Santé Publique”. Given that the tissue samples are classed as surgical waste, that they were used anonymously (the patient's identity is inaccessible to the researchers), and that they were not in any way genetically manipulated, article L. 1211-2 stipulates that their use for research purposes is allowed provided that the patient does not express any opposition to this to the surgeon prior to surgery after being informed of the nature of the research in which they might be potentially employed. Within this framework, the collection and use of this material was furthermore approved by the Institutional Review Board (Comité de Protection des Personnes) of the Centre Hospitalo-Universitaire Pitié-Salpêtrière, Assistance Publique-Hôpitaux de Paris, Paris, France.

All *Macaca mulatta* in this study were captive bred for research purposes. BPRC housing and animal care procedures are in compliance with Dutch law on animal experiments, European directive 86/609/EEC, and with the “Standard for Humane Care and Use of Laboratory Animals by Foreign Institutions”, identification number A5539-01, provided by the Department of Health and Human Services of the US National Institutes of Health (NIH). The local independent ethical committee, constituted according to Dutch law on animal experiments, approved the study protocols prior to start of the experiments. Adult cynomolgus macaques (*Macaca fascicualris*) were imported from Mauritius and housed in the facilities of the Centre d'Energie Atomique (CEA) (Fontenay-aux-Roses, France). Non-human primates (NHP, that includes *M. fascicularis*) are used at the CEA in accordance with French national regulation and under the inspections of national veterinary inspectors. The CEA is in compliance with Standards for Human Care and Use of Laboratory Animals (Animal Welfare Assurance, OLAW number #A5826-01). The use of NHP at CEA is in accordance with recommendation of the Weatherall report as follows: NHP are used at CEA only when no other models (*in vitro* or *in vivo*) are suitable for the aims of the project (recommendation n°1); the study is in the field of infectious diseases (recommendation n°2), *Plasmodium* physio-pathology; NHP are breed following the recommendation of the ETS123 and in accordance with the newly published Eureopean Directive (2010/63) (recommendation n°9). It should be stressed that none of the animal were specifically used for this work, since the liver fragments were collected at necropsy from animals that were euthanized in the course of unrelated studies, thus no suffering was specifically associated with the surgical procedure to obtain the liver fragments. This approach is fully in accordance with the 3R and reduces the number of animal used. Animal suffering avoidance and refinement of procedures are included in CEA aims. The animals were used under the supervision of the veterinarians in charge of the animal facility and the protocols employed were reviewed by the Ethical Animal Committee of the CEA. During ethical review, the end points and analgesia were reviewed and optimised. Experimental procedures were conducted in strict accordance with the recommendations of the European guidelines for the care and use of laboratory animals (European directive 86/609/EEC, OJ, L358, December 18, 1986). In addition, the CEA facilities was accredited (assurance identification number #A5826-01) by the National Institute of Health (USA) Office for Laboratory Animal Welfare (OLAW). The protocols and the use of hepatocytes for the purpose of the work described herein were approved by the Ethical Animal Committee of the CEA (Permit Number A 92-032-02).

### Drugs

Primaquine, 8-(4-amino-1-methylbutylamino)-6-methoxyquinoline diphosphate salt, and pyrimethamine, 5-(4-chlorophenyl)-6-ethyl-2,4-pyrimidinediamine, were purchased from Sigma-Aldrich St Louis, MO, USA (P9504 and P771, respectively); atovaquone, *trans*-2-[4-(4-chlorophenyl)cyclohexyl]-3-hydroxy-1,4-naphthalenedione was purchased from Laboratoire GlaxoSmithKline, France.

### Sporozoites

*P. falciparum* (strain NF54) sporozoites were obtained from infected *Anopheles stephensi* salivary glands collected on days 14–21 after an infective blood meal on a membrane-based feeder system (Department of Medical Microbiology, University Medical Centre St. Radboud, Nijmegen, The Netherlands). *Plasmodium cynomolgi* (M strain) sporozoites were obtained from infected *Anopheles stephensi* salivary glands collected on days 14–35 after an infective blood meal on blood from a *Macaca mulatta*, infected with blood stage *P. cynomolgi* parasites using membrane-based glass feeders (Biomedical Primate Research Centre, Rijswijk, The Netherlands).

### Primary hepatocytes

Human hepatocytes were isolated from liver segments taken from adult patients in the course of partial hepatectomy (Service de Chirurgie Digestive, Hépato-Bilio-Pancréatique et Transplantation Hépatique, Hôpital Pitié-Salpêtrière, Paris, France). Simian hepatocytes were isolated from *Macaca fascicularis* or *M. mulatta* liver segments (provided by Roger Le Grand, commissariat à l'Energie Atomique CEA, Fontenay-aux-Roses, France, or by Clemens H M Kocken, Biomedical Primate Research Centre, Rijswijk, The Netherlands, respectively) taken from healthy animals. The primary hepatocytes were isolated using two-step enzymatic perfusion as previously described [26]. Briefly, the hepatic segments were successively perfused with HBSS-HEPES buffer and with collagenase (C5138, Sigma-Aldrich St Louis, MO, USA), and then dissociated. Viable cells were recovered after centrifugation over a 37% Percoll layer. The isolated simian primary hepatocytes that were obtained were immediately cryopreserved. On the days when human liver segments became available, and when the viability of the primary hepatocytes isolated from these was deemed satisfactory for infection with parasites, an aliquot of the *M. fascicularis* primary hepatocytes was thawed and the two host cell types were separately seeded and incubated for 24 h before infection.

The primary hepatocytes were seeded at a density of 8.3×10^4^ cells per well allowing cell confluence in µclear plate Black 96 wells (Greiner Bio-one, France), or 2.1×10^5^ cells per well in Lab-Tek 8-chambers slides, in both cases coated with rat tail collagen I (Becton Dickinson, Le Pont de Claix, France) in complete medium: William's medium E (Gibco, Cergy-Pontoise, France) supplemented with 10% foetal calf serum (Perbio), 5×10^−5^ M hydrocortisone hemisuccinate (Upjohn SERB Laboratoire), 5 µg/ml Insulin (Sigma), 2 mM L-Glutamine (25030-024, Gibco), 0.02 U/ml–0.02 µg/ml Penicillin-streptomycin (15140-122, Life Technologies) and incubated at 37°C, 5% CO2 for 24 h before infection.

### Pre-erythrocytic cultures and drug assays

After isolation, the *P. falciparum* or *P. cynomolgi* sporozoites were suspended in complete medium and added to their respective host's primary hepatocyte cultures. For drug assays, 3×10^4^ sporozoites were added to each well, or 1×10^5^ *P. cynomolgi* sporozoites for cultures initiated in Lab-Tek 8-chambers slides. Infected cultures were fixed with cold 100% methanol on Days 1, 3, 5, 7, 9 and 11 post-infection in order to count and measure the size of the *P. cynomolgi* or *P. falciparum* pre-erythrocytic (PE) forms obtained. For the drug assays, the antimalarial drugs were added at different concentrations to complete medium. Infected cells were exposed to the drug for 3 days from Day 5 until fixation at Day 8 post-infection. In a second set of experiments, PE-uni selection was included by treatment of *P. cynomolgi* hepatic cultures with atovaquone at 67 ng/ml (182 nM) between Day 5 and Day 8. This was followed by a 2 days treatment with either the same concentration of ATO or with PQ at 10 µg/ml (22 nM), until fixation at Day 10 post-infection. In all cases, the medium containing the drug was renewed daily during treatment. Assays for each drug were generally conducted in two or three independent experiments. In an individual experiment, each concentration was tested in duplicate or triplicate wells.

Hepatic parasites were enumerated by immunofluorescence analysis. Briefly, following fixation, the parasites were specifically labelled with a mouse polyclonal serum raised against the *P. falciparum* heat shock protein 70.1 (PfHSP70.1) obtained after immunization with the recombinant protein. This serum also cross-reacts with *P. cynomolgi* HSP70. The labelled parasites were visualized with Alexa 488-conjugated goat anti-mouse immunoglobulin (Invitrogen). Parasite and host cell nuclei were stained with 1 µg/ml of diamidino-phenylindole (DAPI; Sigma). Parasites were enumerated by examination of the cultures under a fluorescence microscope at 200× magnification (Leica DMI 4000 B). Photomicrographs were obtained using a confocal Olympus BX61 microscope at 600× magnification on cells cultures in Lab-Tek 8-chambers slides.

The cytotoxicity of the anti-malarial drugs was evaluated on *M. fascicularis* hepatocytes treated as for the drug activity assays, through the colorimetric methylthiazolyldiphenyl-tetrazolium bromide test (MTT) [27]. Briefly, the primary hepatocytes were exposed to various concentrations of drug and three days later, a 100 µl MTT solution (500 µg/mL) was added in each well. After 4 h of incubation at 37°C, the plates were read in a spectrophotometer (540 nm absorbance wavelength) and the results were expressed as percentage of cellular viability compared to the non-treated control.

### Data analysis

Dose-response curves and inhibitory concentrations 50% (IC_50_) were calculated by non-linear regression analysis using GraphPad Prism software with the data previously normalized to the untreated controls. Mean parasite numbers were compared using the Mann-Whitney non-parametric test. A *p*-value of less than 0.05 was considered to be statistically significant in the tests.
